# Valproic Acid Neuroprotection in the 6-OHDA Model of Parkinson's Disease Is Possibly Related to Its Anti-Inflammatory and HDAC Inhibitory Properties

**DOI:** 10.1155/2015/313702

**Published:** 2015-02-19

**Authors:** José Christian Machado Ximenes, Kelly Rose Tavares Neves, Luzia Kalyne A. M. Leal, Marta Regina Santos do Carmo, Gerly Anne de Castro Brito, Maria da Graça Naffah-Mazzacoratti, Ésper Abrão Cavalheiro, Glauce Socorro de Barros Viana

**Affiliations:** ^1^Faculty of Medicine Estácio of Juazeiro do Norte, Avenida Tenente Raimundo Rocha 515, 63048-080 Juazeiro do Norte, CE, Brazil; ^2^Faculty of Medicine of the Federal University of Ceará, Rua Coronel Nunes de Melo 1127, 60430-270 Fortaleza, CE, Brazil; ^3^School of Medicine of the Federal University of São Paulo, Rua Botucatu 862, 04023-900 São Paulo, SP, Brazil

## Abstract

Parkinson's disease is a neurodegenerative disorder where the main hallmark is the dopaminergic neuronal loss. Besides motor symptoms, PD also causes cognitive decline. Although current therapies focus on the restoration of dopamine levels in the striatum, prevention or disease-modifying therapies are urgently needed. Valproic acid (VA) is a wide spectrum antiepileptic drug, exerting many biochemical and physiological effects. It has been shown to inhibit histone deacetylase which seems to be associated with the drug neuroprotective action. The objectives were to study the neuroprotective properties of VA in a model of Parkinson's disease, consisting in the unilateral striatal injection of the neurotoxin 6-OHDA. For that, male Wistar rats (250 g) were divided into the groups: sham-operated (SO), untreated 6-OHDA-lesioned, and 6-OHDA-lesioned treated with VA (25 or 50 mg/kg). Oral treatments started 24 h after the stereotaxic surgery and continued daily for 2 weeks, when the animals were subjected to behavioral evaluations (apomorphine-induced rotations and open-field tests). Then, they were sacrificed and had their mesencephalon, striatum, and hippocampus dissected for neurochemical (DA and DOPAC determinations), histological (Fluoro-Jade staining), and immunohistochemistry evaluations (TH, OX-42, GFAP, TNF-alpha, and HDAC). The results showed that VA partly reversed behavioral and neurochemical alterations observed in the untreated 6-OHDA-lesioned rats. Besides, VA also decreased neuron degeneration in the striatum and reversed the TH depletion observed in the mesencephalon of the untreated 6-OHDA groups. This neurotoxin increased the OX-42 and GFAP immunoreactivities in the mesencephalon, indicating increased microglia and astrocyte reactivities, respectively, which were reversed by VA. In addition, the immunostainings for TNF-alpha and HDAC demonstrated in the untreated 6-OHDA-lesioned rats were also decreased after VA treatments. These results were observed not only in the CA1 and CA3 subfields of the hippocampus, but also in the temporal cortex. In conclusion, we showed that VA partly reversed the behavioral, neurochemical, histological, and immunohistochemical alterations observed in the untreated 6-OHDA-lesioned animals. These effects are probably related to the drug anti-inflammatory activity and strongly suggest that VA is a potential candidate to be included in translational studies for the treatment of neurodegenerative diseases as PD.

## 1. Introduction

Parkinson's disease (PD) is the second most common neurodegenerative disorder, primarily characterized by bradykinesia, rigidity, resting tremor, and postural instability. These motor signs are mainly due to progressive degeneration of dopaminergic (DA) neurons in the* substantia nigra pars compacta* (SNpc). Furthermore, PD is also associated with nonmotor features such as cognitive deficits, emotional changes, sleep perturbations, sensation disturbances, autonomic dysfunction, and gastrointestinal symptoms [1–3].

The gold standard for the symptomatic therapy of PD is based on the DA replacement by L-DOPA in combination with an inhibitor of its peripheral conversion to DA. Furthermore, long-term treatment with L-DOPA commonly leads to progressive loss of efficacy, the development of dyskinesias, and nonmotor manifestations [4–6]. Thus, considering the adverse effects and limitations of current therapeutic regimens, there is an urgent need for improved pharmacological options [7]. Most importantly, PD is a chronic neurodegenerative disease associated with substantial morbidity, increased mortality, and high economic burden. Thus, effective management of PD can minimize disability and potentially improve long-term outcomes which would decrease health care costs [8].

Valproic acid is a well-established broad-spectrum drug for the treatment of epileptic seizures, as well as mania and bipolar disorders [9]. In the human brain, valproic acid affects GABA function by potentiating the inhibitory activity of this neurotransmitter, through several ways, including the inhibition of GABA degradation, increased synthesis of GABA, and decreased GABA turnover [10–12]. VA was also found to attenuate NMDA-mediated excitation, block voltage-dependent Na^+^ channels, and modulate the firing frequency of neurons [12, 13]. Investigations uncovered the potential of valproic acid to interfere with multiple regulatory mechanisms, including histone deacetylases (HDAC), GSK3-alpha and beta, Akt, the ERK and phosphoinositol pathways, and the tricarboxylic acid cycle, besides GABA and the OXPHOS system [14].

Evidences indicate a neuroprotective action of VA in the rotenone rat model of PD, where the drug reverted the decrease of the dopaminergic marker TH in the* substantia nigra* and striatum, caused by 7-day toxin administration. VA treatment also significantly counteracted the death of nigral neurons and the 50% drop of striatal dopamine levels caused by rotenone administration [15]. These authors also showed that the PD-marker, the native form of alpha-synuclein, decreased in the* substantia nigra* and striatum from rotenone-treated rats, while nonubiquitinated alpha-synuclein increased in the same regions. These alterations were both counteracted by VA.

The same group of investigators [16] demonstrated that chronic VA administration significantly reduced degenerations of dopaminergic neurons in the* substantia nigra* and of dopaminergic terminals in the striatum, in rats subjected to the unilateral lesion of the nigrostriatal pathway. VA treatment was also able to increase alpha-synuclein expression in the* substantia nigra* and to counteract the lesion-dependent decrease of the protein in the* substantia nigra* and striatum. Furthermore, VA is a known histone deacetylase (HDAC) inhibitor [17, 18] and this event could be related to the anti-inflammatory and neuroprotective properties of the drug, as described for other HDAC inhibitors [17, 19, 20].

Thus, the objectives of the present study focused on the neuroprotective action of VA in an experimental model of PD, consisting of a 6-OHDA unilateral striatal injection in rats. The drug behavioral (apomorphine-induced rotations and spontaneous locomotor activity), neurochemical (DA and DOPAC concentrations in the rat striatum), and immunohistochemical (TH, OX-42, GFAP, and HDAC) alterations were evaluated in 6-OHDA-lesioned animals, after VA treatments. Besides, Fluoro-Jade staining was also explored in the tested groups.

## 2. Material and Methods

### 2.1. Drugs

Valproic acid (Depakene, sodium valproate syrup containing 50 mg valproic acid per 1 mL) was purchased from Abbott Laboratories of Brazil (São Paulo, SP). 6-OHDA, apomorphine, and standard monoamines were from Sigma-Aldrich (St Louis, MO, USA). Ketamine (50 mg/mL) and xylazine (20 mg/mL) were from König (Santana de Parnaíba, São Paulo, Brazil). Antibodies for immunohistochemical assays were from Santa Cruz Biotechnology (Dallas, TX, USA) or Merck-Millipore (Darmstadt, Germany). All other reagents were of analytical grade.

### 2.2. Animals

Male Wistar rats (200–250 g) were maintained at a temperature of 24 ± 2°C, in a 12 h dark/12 h light cycle, with standard food and water* ad libitum*. The study was approved by the Ethical Committee for Animal Experimentation of the Faculty of Medicine of the Federal University of Ceará (Brazil). All experiments followed the ethical principles established in the Guide for the Care and Use of Laboratory Animals, USA, 1986.

### 2.3. Experimental Protocol

The animals were anesthetized with an association of xylazine (10 mg/kg, i.p.) and ketamine (80 mg/kg, i.p.), submitted to trichotomy of the head superior region, and fixed to the stereotaxic frame by their ear canals. Then, a longitudinal midline incision was done and the tissues were separated for bregma visualization. The following coordinates (at two different points) were used: 1st point (AP, +0.5; ML, −2.5; DV, +5.0) and 2nd point (AP, −0.9; ML, −3.7; DV, +6.5). Then, a thin hole was performed in the skull, over the target area, and a 1 *μ*L solution containing 6 *μ*g 6-OHDA was injected into each point. The syringe stayed in place for 5 min to assure the solution diffusion, and then the incision was sutured. The sham-operated (SO) animals were subjected to all procedures, except that saline was injected into the two points. Afterwards, the animals returned to their cages for recovering. They were divided into the following groups: SO (sham-operated, treated with distilled water), 6-OHDA-lesioned (also treated with distilled water), 6-OHDA-lesioned + VA25, and 6-OHDA-lesioned + VA50. The treatments started 24 h after the surgical procedure and all groups were treated orally and daily for 15 days (0.2 mL/100 g body weight). Then, after treatments and 1 h after the last drug administration, the animals were submitted to behavioral tests. After that, they were euthanized (decapitation) and brain tissues were removed for neurochemical, histological, and immunohistochemical studies.

### 2.4. Behavioral Testing

#### 2.4.1. Apomorphine-Induced Rotations

The contralateral rotation (opposite to the lesioned right-side) induced by apomorphine (1 mg/kg, i.p.) was monitored for 1 h. The cause of this apomorphine-induced rotational behavior is related to the unbalance in the nigrostriatal dopaminergic pathways, between the right (lesioned) and left (unlesioned) brain hemispheres.

#### 2.4.2. Open-Field Test

This test evaluates a stimulant or depressant drug activity and may also indicate an anxiolytic activity. The arena was made of wood, whose dimensions were 50 cm × 50 cm × 30 cm (length, width, and height). The floor was divided into 4 quadrants of equal size. At the time of the experiment, the apparatus was illuminated by a red light. The following parameters were observed for 5 min: number of crossings with the four paws from one quadrant to another (what measures the locomotor spontaneous activity) and number of rearings (stereotyped vertical exploratory movements).

### 2.5. Neurochemical Determinations of DA and DOPAC

The striatal contents of dopamine (DA) and DOPAC (the main brain DA metabolite) were determined by HPLC. Homogenates were prepared in 10% HClO_4_ and centrifuged at 4°C (15,000 rpm, 15 min). The supernatants were filtered and 20 *μ*L were injected into the HPLC column. For that, an electrochemical detector (model L-ECD-6A from Shimadzu, Japan) coupled to a column (Shim-Pak CLC- ODS, 25 cm) with a flux of 0.6 mL/min was employed. A mobile phase was prepared with monohydrated citric acid (150 mM), sodium octyl sulfate (67 mM), 2% tetrahydrofuran, and 4% acetonitrile in deionized water. The mobile phase pH was adjusted to 3.0 with NAOH (10 mM). Monoamines were quantified by comparison with standards which were processed the same manner as the samples. The results are expressed as ng/g wet tissue.

### 2.6. Histological and Immunohistochemistry Analyses in Rat Mesencephalon, Striatum, and Hippocampus

#### 2.6.1. Fluoro-Jade

Fluoro-Jade is an anionic fluorescein derivative, useful for the histological staining of neurons undergoing degeneration. After paraffin removal (by immersion in xylol), tissue sections surrounded by gelatin were mounted on slides. The tissue was rehydrated by immersion in ethanol for 3 min, followed by immersions in 70 and 50% ethanol solutions and distilled water. The slices were transferred into a 0.06% potassium permanganate solution, for 15 min, washed in distilled water, and transferred to a Fluoro-Jade solution where they stayed for 30 min (with gentle stirring). After staining, the slices were washed in distilled water (3 times, 2 min each time). The excess of water was discarded and the dry slices were mounted in a fluoromount medium and examined with a fluorescence microscope.

#### 2.6.2. Immunohistochemistry Assays for OX-42 (CD11b) and GFAP

Microglia are the resident macrophages of the central nervous system and the first line of immune defense cells. Immunohistochemistry assays were used for demonstration of microglia and astrocytes, important glia cells associated with neurodegenerative processes. OX-42 was used as a marker for the presence of microglia, while for astrocytes the GFAP (glial fibrillary acidic protein) marker was used. Slices were washed 3 times with PBS (5 min each), followed by PBS containing 0.2% Triton X-100 and 10% horse serum, for 1 h, at RT. Then, the slices were incubated with primary antibodies prepared in a blockade solution (anti-CD11b, 1 : 100, mouse IgG1, AbD Serotec, or anti-GFAP, 1 : 500, rabbit polyclonal, from Sigma-Aldrich) overnight, at RT. At the next day, the slices were washed 3 times (10 min each) with PBS and incubated for 2 h, at RT, with secondary antibodies (donkey anti-mouse or donkey anti-rabbit) diluted to 1 : 500. The secondary antibodies are conjugated with fluorochromes—Alexa Fluor 594 (red) or Alexa Fluor 488 (green). Finally, the slices were washed 3 times with PBS, counterstained with DAPI (Vector Laboratories, UK) for 10 min, and washed again 3 times with PBS (5 min each). The slices were mounted in silanized slides with the fluorescent medium from Dako (USA) or Fluoromount (Sigma-Aldrich, USA) and kept at dark (−20°C), until visualization in a fluorescent microscope.

#### 2.6.3. Immunohistochemistry Analyses for Tyrosine Hydroxylase (TH), Tumor Necrosis Factor-Alpha (TNF-Alpha), and Histone Deacetylase (HDAC)

Brain striatal sections were fixed in 10% buffered formol, for 24 h, followed by a 70% ethanol solution. The sections were embedded into paraffin wax, for slices processing on appropriate glass slides. These were placed in the oven at 58°C, for 10 min, followed by deparaffinization in xylol, rehydration in ethanol at decreasing concentrations, and washing in distilled water and PBS (0.1 M sodium phosphate buffer, pH 7.2) for 10 min. The endogenous peroxidase was blocked with a 3% hydrogen peroxide solution, followed by incubation with the appropriate primary rabbit polyclonal antibody for TH, TNF-alpha, and HDAC, and diluted according to the manufacturers' instructions (Santa Cruz Biotechnology or Merck-Millipore), for 2 h, at room temperature in a moist chamber. The glass slides were then washed with PBS (3 times, 5 min each) and incubated with the biotinylated secondary antibody, for 1 h, also at room temperature in a moist chamber. Then, they were washed again in PBS and incubated with streptavidin-peroxidase, for 30 min, at room temperature in a moist chamber. After another wash in PBS, they were incubated in 0.1% DAB solution (in 3% hydrogen peroxide). Finally, the glass slides were washed in distilled water and counterstained with Mayer's hematoxylin, washed in tap water, dehydrated in ethanol (at increasing concentrations), diaphanized in xylol, and mounted on Entelan, for optic microscopy examination.

### 2.7. Statistical Analyses

For statistical analysis, one-way ANOVA, followed by Newman-Keuls as the* post hoc* test, was used for multiple comparisons. Whenever needed, the paired or unpaired Student's *t*-tests were used for comparisons between two means. Differences were considered significant at *P* < 0.05.

## 3. Results

### 3.1. Behavioral Evaluation

#### 3.1.1. Valproic Acid (VA) Effects on Apomorphine-Induced Rotational Behavior

**Figure 1:**
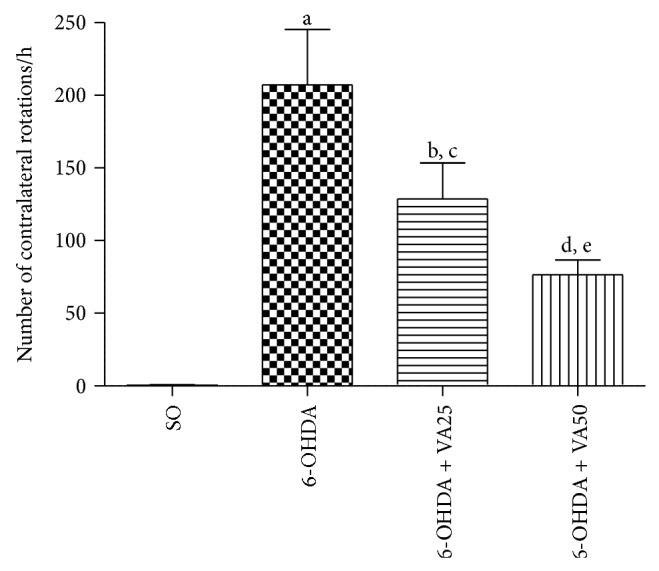
Valproic acid (VA) treatments of the 6-OHDA group decrease the apomorphine-induced contralateral rotations. Values are mean ± SEM from 8–10 animals per group. a. versus SO, *q* = 8.397; b. versus SO, *q* = 5.334; c. versus 6-OHDA, *q* = 3.373; d. versus SO, *q* = 3.162; e. versus 6-OHDA, *q* = 5.616 (one-way ANOVA and Newman-Keuls as the* post hoc* test).

The results showed that the untreated 6-OHDA-lesioned group presented an average of 207.2 contralateral rotations per hour, as related to the SO group which presented almost no behavioral alteration after the apomorphine administration. On the other hand, the apomorphine-induced rotational behavior was significantly reduced in a dose-related manner by 1.6 and 2.7 times, after VA (25 and 50 mg/kg) treatments, respectively, as compared to the untreated 6-OHDA-lesioned group (Figure 1).

#### 3.1.2. Effects of VA on the Locomotor Activity and Rearing Behavior, as Evaluated by the Open-Field Test

**Figure 2:**
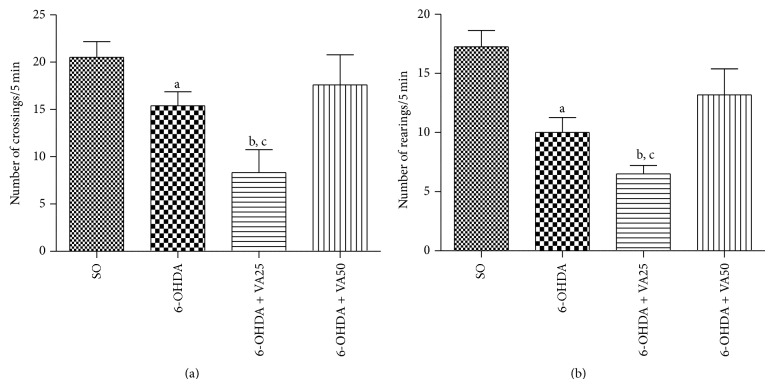
The treatment with valproic acid (VA) reverses the decreased locomotor activity (a) and rearing behavior (b) of the untreated 6-OHDA group, whose values go towards those of the sham-operated control (SO). Values are mean ± SEM from 5–8 animals per group. (a) a. versus SO, *t* = 2.276, df = 14; b. versus SO, *t* = 4.287, df = 12; c. versus 6-OHDA, *t* = 2.611, df = 12. (b) a. versus SO, *t* = 3.841, df = 14; b. versus SO. *t* = 6.167, df = 12; c. versus 6-OHDA50, *t* = 3.132, df = 9 (two-tailed Student's *t*-test).

The number of crossings per 5 min was reduced by 25% and unexpectedly by 60% in the untreated 6-OHDA-lesioned group and in the 6-OHDA-lesioned group treated with VA at the lower dose (25 mg/kg), respectively, as related to the SO group (Figure 2(a)). However, the decreased locomotion of the untreated 6-OHDA-lesioned group was completely reversed after the treatment with the higher VA dose (50 mg/kg). Similar results were observed with the rearing behavior (Figure 2(b)) which was reduced by 42 and 62% in the untreated 6-OHDA and in the 6-OHDA + VA25 groups, respectively, as related to the SO group. This alteration was in part reverted after treatment with the higher VA dose (50 mg/kg).

### 3.2. Neurochemical Evaluation

#### 3.2.1. Effect of VA on Striatal DA and DOPAC Concentrations (ng/g Tissue) in the Model of PD in Rats

**Figure 3:**
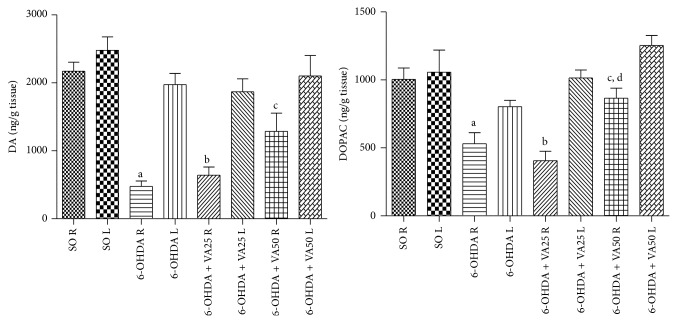
Valproic acid (VA) treatments partly reverse the decrease of dopamine (DA) and DOPAC contents observed in the untreated 6-OHDA group. Values are mean ± SEM from 8–17 animals per group. DA: a. versus left side (L) of the same group, *t* = 7.195, df = 10; b. versus left side (L) of the same group, *t* = 6.874, df = 7; c. versus 6-OHDA, right side (R), *t* = 2.690, df = 22. DOPAC: a. versus left side (L) of the same group, *t* = 3.059, df = 26; b. versus left side (L) of the same group, *t* = 6.671, df = 23; c. versus left side (L) of the same group, *t* = 3.653, df = 16; d. versus 6-OHDA, right side (R), *t* = 2.985, df = 20 (two-tailed Student's *t*-test).

The right lesioned-side of the untreated 6-OHDA group showed 76 and 78% reductions in DA contents, as related to its left side or to the right side of the SO group, respectively. As expected, the SO group presented similar DA values in both sides. Decreases of 66 and 70% were seen in the lesioned right side of the 6-OHDA group, after VA treatments with the lower dose (25 mg/kg), in relation to its left side or to the right side of the SO group, respectively. However, lower reductions in DA contents (39 and 41%) were shown in the lesioned right striatum of the 6-OHDA group after treatments with the higher VA dose (50 mg/kg), as related to its left side or to the right side of the SO group (Figure 3, DA). As far as DOPAC concentrations are concerned, reductions of 34 and 47% (Figure 3, DOPAC) were observed in the right striatum, as related to the left side of the untreated 6-OHDA group or to the right side of the SO group, respectively. On the other hand, similar values of DOPAC were seen in both sides of the striatum in the SO group. Reductions of 60 and 50%, respectively, in DOPAC contents were observed in the right striatum of the 6-OHDA + VA25 group, as related to its left side and to the right side of the untreated 6-OHDA group. However, 31 and only 14% reductions in DOPAC values were observed in the right side of the 6-OHDA group, after treatments with VA at the higher dose (50 mg/kg), as related to the left side of the untreated 6-OHDA group or to the right side of the SO group, respectively.

### 3.3. Histological and Immunohistochemical Analyses of VA Effects in the PD Model in Rats

#### 3.3.1. Histological Evaluation by Fluoro-Jade

**Figure 4:**
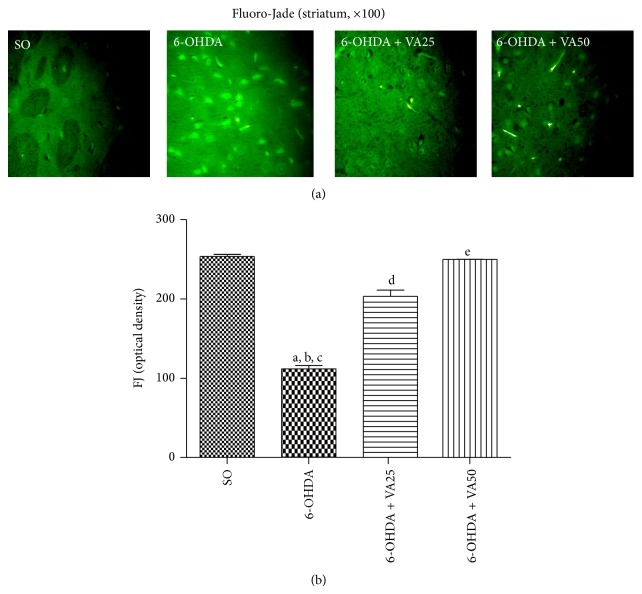
Representative photomicrographs (a) showing that valproic acid (VA) treatments (25 and 50 mg/kg) of the 6-OHDA group reverse the increased Fluoro-Jade staining (seen as a high green fluorescence) in the rat striatum. SO means the sham-operated group, where a low green fluorescence against a dark background is observed, indicating a lesser neuron degeneration (magnification ×100). The Image J software was used to measure the relative density of cells in 3–5 fields (b). a. versus SO, *q* = 30.64; b. versus 6-OHDA + VA25, *q* = 19.95; c. versus 6-OHDA + VA50, *q* = 30.15; d. versus SO, *q* = 10.69; e. versus 6-OHDA + VA25, *q* = 10.20 (one-way ANOVA and Newman-Keuls as the* post hoc* test).

Photomicrographs in Figure 4 show a greater number of Fluoro-Jade stained degenerating neurons in the striatum that appear bright green, in the untreated 6-OHDA-lesioned group. This profile changed towards a darker background, indicative of less neuronal degeneration in the 6-OHDA-lesioned groups, after VA treatments (25 and 50 mg/kg), similarly to that observed in the SO group. The histogram represents the relative optical density of cells, measured in 3 to 5 fields by the Image J software.

#### 3.3.2. VA Effects on the Tyrosine Hydroxylase (TH) Immunoreactivity

**Figure 5:**
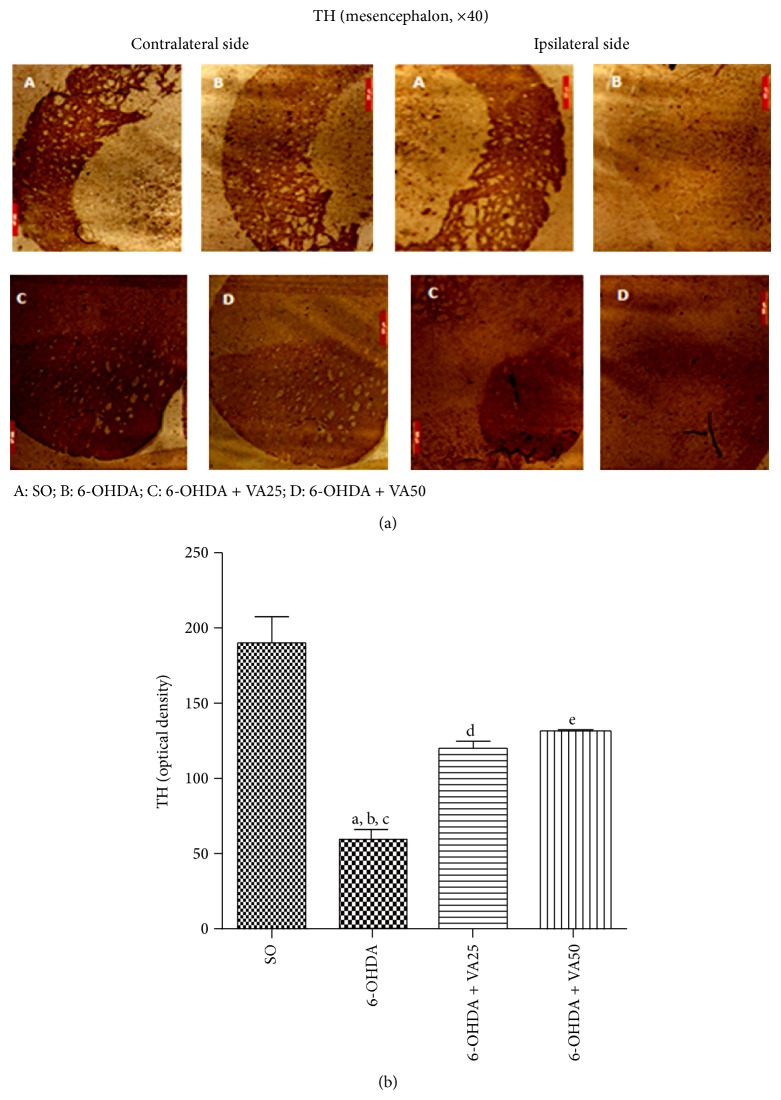
Representative photomicrographs (a) showing that the valproic acid (VA) treatment (50 mg/kg) reverses the decreased immunoreactivity for tyrosine hydroxylase (TH) of the 6-OHDA group, in the rat mesencephalon lesioned (ipsilateral) side. SO means the sham-operated group, where a high immunoreactivity for TH is observed in both the contralateral (cont., unlesioned) and ipsilateral (ips., lesioned) sides. Scale bars represent 500 *μ*m (magnification ×400). Measurements (b) by Image J software of relative optical densities from 3–5 fields: a. versus SO, *q* = 12.72; b. versus 6-OHDA + VA25, *q* = 5.51; c. versus 6-OHDA + VA50, *q* = 6.48; d. versus SO, *q* = 6.26; e. versus SO, *q* = 5.29 (one-way ANOVA and Newman-Keuls as the* post hoc* test).

A biochemical abnormality present in PD is the degeneration of dopaminergic neurons in the* substantia nigra pars compacta*, resulting in the reduction of dopamine contents. Since TH catalyzes the formation of L-dihydroxyphenylalanine (L-DOPA), limiting step in DA biosynthesis, PD may be considered a striatal TH deficiency syndrome. Thus, this enzyme is considered as a biomarker in PD models. In the present work, although there was a decrease in the number of TH immunopositive cells, in the left side of the mesencephalon, this decrease was much higher in the right lesioned side. On the other hand, the right sides of 6-OHDA groups after treatments with VA, at the dose of 50 mg/kg, presented a lower reduction in the TH immunoreactivity, as related to the right side of the untreated 6-OHDA group, indicating attenuation of the 6-OHDA neurotoxicity (Figure 5(a)). The results were quantified by the Image J software and shown as histograms of relative optical densities (Figure 5(b)).

#### 3.3.3. VA Effects on the Immunoreactivity for OX-42 and GFAP

**Figure 6:**
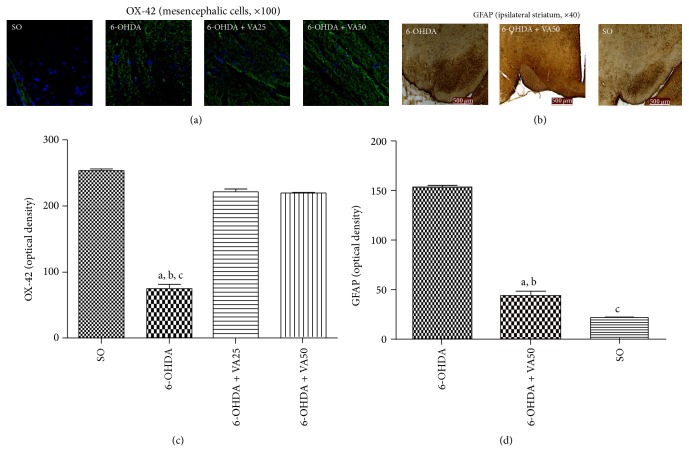
Representative photomicrographs ((a), (c)) and measurements of corresponding relative optical densities in 3–5 fields ((b), (d)), showing that valproic acid (VA) treatments reverse the increases of OX-42, visualized as a green fluorescence with cells nuclei presenting a blue fluorescence, and of GFAP immunoreactivities (brown staining), in the rat mesencephalon (right lesioned side) of the untreated 6-OHDA group. Scale bars represent 500 *μ*m (magnifications: ×100 for OX-42 and ×40 for GFAP). The relative optical densities were measured by Image J software. OX-42: a. versus SO, *q* = 47.94; b. versus 6-OHDA + VA25, *q* = 41.46; c. versus 6-OHDA + VA50, *q* = 40.90. GFAP: a. versus SO, *q* = 27.40; b. versus 6-OHDA + VA50, *q* = 23.11; c. versus SO, *q* = 4.29 (one-way ANOVA and Newman-Keuls as the* post hoc* test).

Microglia are the immune effector cells of the CNS. The reactive cell form represents a population of macrophages, which are associated with brain injury and neuroinflammation. Microglia are considered the most potent antigen presenting cells, in the CNS. Like macrophages, reactive microglia secrete a number of inflammatory mediators, which serve to orchestrate the cerebral immune response. Similarly, astrocytes constitute an important cellular population within the CNS and contribute to the normal function of this system. These cells express the glial fibrillary acidic protein (GFAP), important for their morphology and movement control, besides being involved in astrocyte-neuron interactions. Thus, astrocyte processes mediated by GFAP exert a fundamental role in the synaptic efficacy modulation, in the CNS. The reason for the specific loss of dopaminergic neurons in the SNpc in PD may be related to astrocyte properties in this area [21]. Our photomicrograph data showed higher OX-42 immunostaining in the right mesencephalon of the untreated 6-OHDA-lesioned rats, as related to those of the 6-OHDA groups after VA treatments (25 and 50 mg/kg). As expected, less OX-42 immunostaining was visualized in the SO group, suggesting the presence of a lower number of activated microglia. The results were quantified by the Image J software (Figure 6(b)). Similar results were observed in the case of GFAP immunostaining, where a great number of immunostained cells were demonstrated in the untreated 6-OHDA-lesioned group, as related to the lesioned group after VA treatment (50 mg/kg) and to the SO group (Figure 6(d)).

#### 3.3.4. Immunohistochemistry for TNF-Alpha

**Figure 7:**
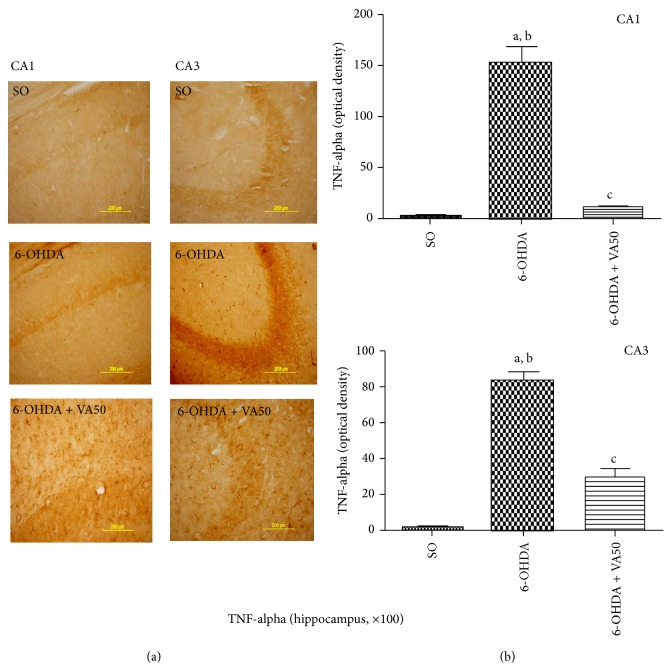
Representative photomicrographs (a) showing that valproic acid (VA) treatments reverse the increased TNF-alpha immunoreactivities of the 6-OHDA group in CA1 and CA3 hippocampus subfields. Scale bars represent 200 *μ*m (magnification ×100). In (b), histograms are shown with relative optical densities measured with the Image J software. CA1: a. versus SO, *q* = 19.51; b. versus 6-OHDA + VA50, *q* = 18.24; c. versus SO, *t* = 6.33, df = 4. CA3: a. versus SO, *q* = 25.38; b. versus 6-OHDA + VA50, *q* = 8.66; c. versus 6-OHDA, *q* = 16.72 (one-way ANOVA and Newman-Keuls as the* post hoc test*, and unpaired Student's *t*-test).

We showed an upregulation of TNF-alpha, expressed as a higher immunoreactivity in CA1 and CA3 areas of the hippocampus (Figure 7(a)) of untreated 6-OHDA-lesioned rats. These effects were reversed in the 6-OHDA-lesioned groups, after VA treatment (50 mg/kg), and the profile was similar to that observed with the SO group.

#### 3.3.5. Immunohistochemistry for Histone Deacetylase (HDAC)

**Figure 8:**
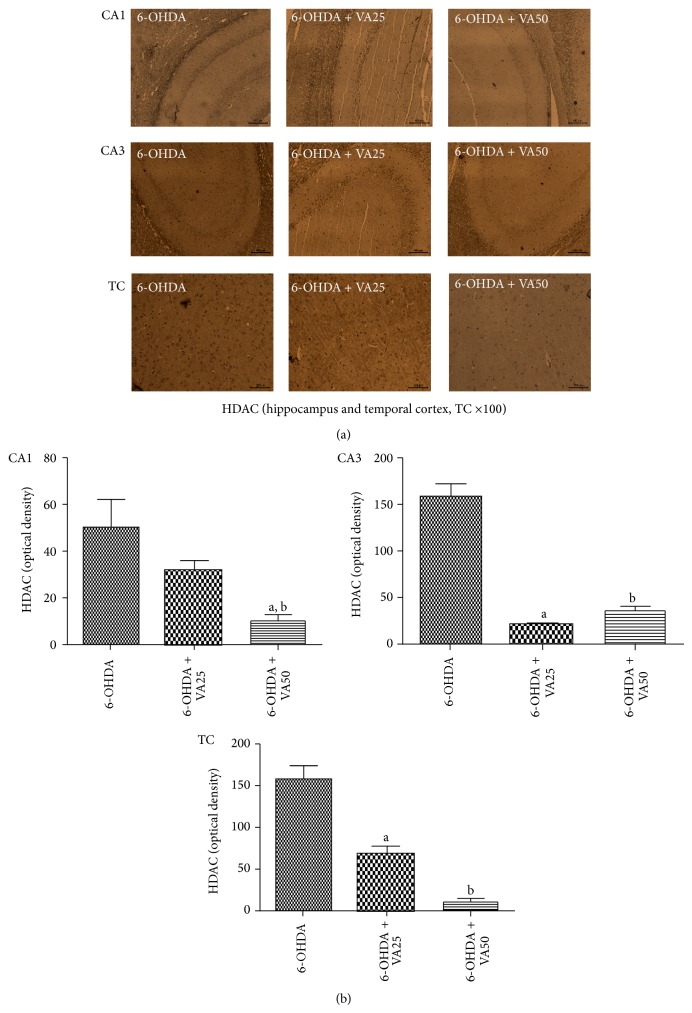
Representative photomicrographs (a) showing that valproic acid (VA) treatments (25 and 50 mg/kg) reverse the histone deacetylase (HDAC) immunoreactivities in CA1 and CA3 hippocampal areas and in the temporal cortex, TC. Scale bars represent 200 *μ*m (magnification ×100). (b) Histograms showing relative optical densities measured by the Image J software. CA1: a. versus 6-OHDA, *q* = 5.65; b. versus 6-OHDA + VA25, *t* = 4.81, df = 8. CA3: a. versus 6-OHDA, *q* = 18.11; b. versus 6-OHDA, *q* = 16.40. TC: a. versus 6-OHDA, *q* = 4.85; b. versus 6-OHDA, *q* = 12.34 (one-way ANOVA and Newman-Keuls as the* post hoc* test and unpaired Student's *t*-test).

A higher immunoreactivity for HDAC was demonstrated in the hippocampus of the untreated 6-OHDA-lesioned rats. This effect occurred mainly in the CA1 and CA3 areas, but also in the temporal cortex. A much lower immunoreactivity was noticed in 6-OHDA-lesioned rats, after treatments with VA at both doses, directing the profile towards that of the SO group, where almost no immunoreactivity was noticed (Figure 8).

## 4. Discussion

Valproic acid (VA) is widely used in clinics as an anticonvulsant/antiepileptic drug and, recently, in the therapy of bipolar disorders and migraine prophylaxis. As an antiepileptics drug, it is considered a drug of wide spectrum, possessing a multiplicity of molecular targets, besides its effects on GABAergic/glutamatergic neurotransmissions and on the modulation of intracellular signaling pathways [22]. Evidences [23–25] have indicated the neuroprotective effects of VA on several* in vivo* experimental models. However, studies on the association of VA anti-inflammatory and antioxidant properties with its neuroprotective actions in experimental models of degenerative disorders, as Parkinson's disease, are relatively few [16, 24, 26].

In the present work, the effects of VA were evaluated in the experimental model of PD, consisting of a unilateral striatal injection of the 6-OHDA neurotoxin in rats. An attempt was made to correlate the VA neuroprotective action to its anti-inflammatory effects and these with neurodegenerative diseases, mainly PD, are already observed by us and others [27–32].

Animal models are important tools for making possible the investigation of pathophysiological mechanisms and therapeutic strategies which are eventually translated to the clinics. The model based on the 6-OHDA striatal lesion is largely used in experimental studies of PD [33–35]. 6-OHDA is a highly specific neurotoxin whose brain targets are catecholaminergic neurons and the dopamine transporter (DAT). This neurotoxin causes an extensive and irreversible loss of dopaminergic neurons in the mesencephalon that is associated with behavioral deficits. However, the importance of experimental models is limited and frequently the results cannot be directly extrapolated to the clinics [36].

Previous studies [37] showed that 6-OHDA acts by two independent manners: the formation of free radicals and inhibition of complexes I and IV of the mitochondrial respiratory chain. The 6-OHDA inhibition of respiratory enzymes is reversible and insensitive to free radicals scavenging and iron chelating drugs, with the exception of deferoxamine. Later [38], alpha-synuclein isoforms were shown to increase cell vulnerability to several insults and 6-OHDA toxicity mediated by DAT. These investigators suggest that the 6-OHDA-induced mechanism of dopaminergic toxicity, evaluated in HEK-293 kidney cells from human embryos, involves interaction of the mutant alpha-synuclein with DAT and the subsequent acceleration of energy depletion, an event that may be relevant to the pathogenesis of PD.

In the model of unilateral striatal injection of 6-OHDA, we observed that this neurotoxin increased by more than 400-fold the apomorphine-induced rotational behavior, indicative of a dopaminergic loss, and this effect was reverted after VA treatments (25 and 50 mg/kg), in a dose-dependent manner. It is known that the 6-OHDA lesion produces a stereotyped behavior, evidenced by the increase of contralateral rotations induced by apomorphine that are manifested around 2 weeks after the establishment of the lesion [39, 40]. Thus, our results suggest a neuroprotective action for VA, in this PD model.

The 6-OHDA-induced striatal lesion produces alterations in the locomotor activity, as evaluated by the open-field test [41]. We showed that untreated 6-OHDA-lesioned animals presented a significant reduction in the number of crossings/5 min, as related to the SO group, and such behavioral alteration was reversed after treatment with the higher VA dose. Unexpectedly, the 6-OHDA-lesioned group treated with the lower VA dose showed an even larger decreased locomotor activity, as related to the untreated 6-OHDA group. Evidences [42–44] show that VA upregulates melatonin MT1 and MT2 receptors, while others [45] demonstrated that VA reverses and prevents amphetamine-induced hyperactivity in an animal model of mania. Melatonin is known to present, on one hand, antidopaminergic activity by interfering with DA release and, on the other hand, presents a neuroprotective action due to its antioxidant activity. Thus, VA presents several effects by interacting with several neurotransmitters, resulting in outcomes not dose-related. Furthermore, VA results, in the apomorphine-induced rotation test, are probably not related to those seen in the open-field test. Similar data were observed in the rearing behavior, suggesting that VA reverses in great part the 6-OHDA-induced behavioral changes. This stereotyped behavior is the result of an extensive loss of dopaminergic neurons in the striatal lesioned area [39, 40, 46–48].

Dopaminergic cell bodies in the SN provide dopaminergic innervations to the striatum, and degeneration of these neurons results in dopamine depletion in that area. In turn, dopamine depletion and the loss of dopamine neurons lead to the hallmark motor dysfunction of PD [21]. In the present study, we showed that the substantial decrease in DA contents, in the striatal lesioned side of the untreated 6-OHDA group, was partly reversed in the 6-OHDA group after VA treatment at the higher dose (50 mg/kg). This effect suggests a neuroprotective action for VA and its potential for the treatment of neurodegenerative diseases as PD. A similar result was observed for DOPAC, the main DA metabolite in the brain. Furthermore, the striatal dopaminergic loss, characteristic of neuron degeneration, was demonstrated in the lesioned right side of the untreated 6-OHDA group, as evaluated by Fluoro-Jade staining. A lesser neuron degeneration was noticed in the 6-OHDA-lesioned group, after treatments with VA at both doses. Most symptoms of PD are the consequence of preferential degeneration of dopamine-synthesizing cells of the mesostriatal-mesocortical neuronal pathway.

In humans, mesencephalic dopamine neurons of the* substantia nigra* and ventral tegmental area are characterized by the presence of protein molecules, as tyrosine hydroxylase, aromatic amino acid decarboxylase, monoamine oxidase, vesicular monoamine transporter, and dopamine transporter, among others, not found in other dopamine-containing neurons of the vertebrate brain [49]. Mitochondrial fragmentation has been shown to be an early event, during apoptosis, and is implicated in the degeneration of DA neurons in PD. Thus, the prevention of mitochondrial fragmentation could rescue cell death in several PD models [50].

A neurochemical abnormality consistent to PD is the degeneration of dopaminergic neurons in the SNpc, leading to reduction of DA contents in the striatum. Since TH catalyzes the formation of DOPAC, limiting step in DA biosynthesis, PD is considered a striatal TH deficit syndrome. Problems related to PD are exacerbated when the vesicular stocks of DA are altered in the presence of alpha-synuclein or oxidative stress [51]. Biochemical* postmortem* studies revealed that the main PD symptoms are caused by DA deficiency in degenerated nigrostriatal dopaminergic terminals. Considering that TH is a limiting enzyme for DA biosynthesis, it plays an important role in PD development. DA levels regulated by TH activity are believed to interact with the alpha-synuclein protein, resulting in intracellular aggregates known as Lewy bodies and apoptotic cell death [52].

A recent study [53] performed with a PD model similar to ours showed that while the detrimental effect of 6-OHDA on the TH+ fibres in the striatum was immediate, the loss of TH+ dendritic fibres and the reduction in cell size and intensity of TH expression, as well as the eventual reduction in the number of TH+ neurons in the* substantia nigra*, were delayed for several days after surgery. In the present work, we showed that while almost no immunoreactivity for TH was observed in the mesencephalon ipsilateral lesioned side of the untreated 6-OHDA group, this effect was partially reversed in the ipsilateral side of the 6-OHDA-lesioned group after VA treatment, suggesting a neuroprotection and potential use of VA in PD treatment.

The presence of reactive microglia was already detected in the* substantia nigra* of patients with neurodegenerative diseases, including PD, almost three decades ago [54], and later the involvement of microglia in neurodegenerative processes such as those of PD [55] was also shown. Others indicated that an inflammatory process in the* substantia nigra*, characterized by activation of microglia, probably initiates or aggravates nigral degeneration in PD [56]. Furthermore, chronic inflammation mediated by microglial cells is considered to be a fundamental process contributing to death of dopaminergic neurons in the brain, and the production of inflammatory agents by those cells characterizes the slow neurodegeneration seen in PD [57]. More recent studies [58] showed that the activation of microglia by LPS, in a mice model, induces PD-like pathogenesis and symptoms which mimic the progressive changes of this pathology. Interestingly, not only microglia but also astrocytes seem to be responsible for the progression of PD, playing an important role in initiating the early tissue response [59].

In the present work, we demonstrated by immunohistochemistry analyses that the treatment of the 6-OHDA-lesioned groups by VA decreased the number of immunopositive cells to OX-42 and GFAP, in the rat mesencephalon, as related to that of the untreated 6-OHDA group. These results indicate that one way by which VA exerts its neuroprotective action may be by decreasing glial cells activation in the brain. In primary neuron-glia cultures from rat midbrain, VA was demonstrated to be a potent neuroprotective drug against LPS-induced neurotoxicity, reducing the release of proinflammatory factors [60]. Others [61] showed that VA protects midbrain DA neurons from LPS or MPTP-induced neurotoxicity, identifying astrocytes as a novel VA target and a potential new role of interactions between DA neurons and astrocytes. All these data confirm our results further, concerning the neuroprotective effects of VA.

Although TNF-alpha is able to exert both homeostatic and pathophysiological roles in the CNS, evidences indicate that, in pathological conditions, microglia release large amounts of TNF-alpha, an important component of the neuroinflammatory response associated to neurological disorders, as Parkinson's disease [62]. Furthermore, VA has been shown to significantly inhibit LPS-induced production of TNF-alpha and IL-6 by human monocytic leukemia and glioma cells [63]. In a previous study, we also demonstrated [27] that VA reduced TNF-alpha immunostaining in carrageenan-inflamed rat paws. In addition, the anti-inflammatory action of VA was potentiated by pentoxifylline, a phosphodiesterase inhibitor known to inhibit the TNF-alpha production. In the present study, we showed that VA treatment of the 6-OHDA-lesioned rats decreased the immunostaining for TNF-alpha, mainly in the CA1 and CA3 hippocampal areas, as related to the untreated group.

PD patients, at an early stage of the disease, show hippocampal and prefrontal atrophy, and impaired memory is related to hippocampal atrophy [64, 65]. According to the authors, these findings suggest that striatal dopaminergic depletion and global brain volume loss contribute to cognitive impairment in nondemented PD patients, but the dysfunctions of extrastriatal dopaminergic or nondopaminergic systems probably play a role, particularly in more generalized cognitive impairments. More recently [66], investigations indicate that learning deficits are associated with volume loss in hippocampal subfields that act as input regions in the hippocampal circuit, suggesting that degeneration in these regions could be responsible for cognitive dysfunction in PD.

Furthermore, clinical and experimental findings support the view that the hippocampus is also implicated in cognitive dysfunctions seen in patients with PD. Moreover, other data suggest interactions between dopaminergic systems and the hippocampus, in synaptic plasticity, adaptive memory, and motivated behavior [67]. Interestingly, new evidence [68] using a model similar to ours shows that the partial dopamine depletion leads to impairment of long term recognition memory, accompanied by abnormal synaptic plasticity in the dentate gyrus, what agrees to our findings.

A large body of evidence suggests that HDACi are neuroprotective drugs and potential candidates for the treatment of neurodegenerative diseases as PD. Thus, the suberoylanilide hydroxamic acid, a histone deacetylase inhibitor, was shown to protect dopaminergic neurons from neurotoxin-induced damage [69]. Others [16] demonstrated that VA, a drug known to present HDAC inhibitory properties, exerts a neuroprotective effect on the rotenone rat model of nigrostriatal degeneration which is similar to ours and in brain ischemia as well [70]. Recently [71], we also observed, for the first time, that caffeine neuroprotection in PD is probably related to its histone deacetylase inhibition. Our data agree with other [24] results, demonstrating that VA was able to partially prevent striatal dopamine depletion and to protect against* substantia nigra* dopaminergic loss in the MPTP mouse model of PD. These data, as ours, suggest that VA may be a potential disease-modifying therapy for PD.

In conclusion, we demonstrated in the present work the neuroprotective action of VA in the 6-OHDA model of PD in rats. The drug partly reversed the behavioral and neurochemical alterations induced by 6-OHDA and also decreased neuron degeneration observed in the striatum of the untreated 6-OHDA lesioned rats. In addition, VA treatment increased TH immunostaining and decreased microglia and astrocyte reactivities and also TNF-alpha, as well as HDAC immunostaining. These effects are probably related to the drug anti-inflammatory activity and strongly suggest VA as a potential candidate to be included in translational studies for the treatment of neurodegenerative diseases as PD.
